# Anatomy of the aortic segmental arteries—the fundamentals of preventing spinal cord ischemia in aortic aneurysm repair

**DOI:** 10.3389/fcvm.2024.1475084

**Published:** 2024-12-03

**Authors:** Paata Pruidze, Jeremias T. Weninger, Giorgi Didava, Karoline M. Schwendt, Stefan H. Geyer, Christoph Neumayer, Josif Nanobachvili, Wolf Eilenberg, Martin Czerny, Wolfgang J. Weninger

**Affiliations:** ^1^ Division of Anatomy, Medical University of Vienna, Vienna, Austria; ^2^ Division of Vascular Surgery, Department of General Surgery, Medical University of Vienna, Vienna, Austria; ^3^ Department of Cardiovascular Surgery, University Heart Center Freiburg, Bad Krozingen, Germany; ^4^ Faculty of Medicine, Albert Ludwig University Freiburg, Freiburg, Germany

**Keywords:** CT, aorta, aortic aneurysm, ASA, aortic segmental arteries, prevention

## Abstract

**Objective:**

Spinal cord ischemia due to damage or occlusion of the orifices of aortic segmental arteries (ASA) is a serious complication of open and endovascular aortic repair. Our study aims to provide detailed descriptions of the proximal course of the ASAs and metric information on their origins.

**Materials and methods:**

Initially, 200 randomly selected, embalmed cadavers of human body donors were anatomically dissected and systematically examined. On macroscopic inspection, 47 showed severe pathologies and were excluded. Of the remaining 153, 73 were males and 80 females.

**Results:**

In total, 69.9% of the aortae showed 26–28 ASA orifices. In 59.5% the most proximal ASA, at least unilaterally, was the third posterior intercostal artery, which originated from the descending aorta at approximately 10% of its length. In 56.2%, the left and right ASAs had a common origin in at least one body segment. This mainly affected the abdominal aorta and L4 in particular (54.2%). The ASAs of lumber segments 1–3 originated strictly segmentally. In contrast, in 80.4%, at least one posterior intercostal artery originated from a cranially or caudally located ipsilateral ASA. Such an arrangement was seen along the entire thoracic aorta. Further descriptions of variants and metric data on ASA orifices are presented.

**Conclusion:**

Our large-scale study presents a detailed topographic map of ASAs. It underscores the value of preoperative CT councils and provides crucial information for interpreting the results. Furthermore, it aids in planning and conducting safe aortic intervention and assists in deciding on single- or two-staged stent graft procedures.

## 1 Introduction

The incidence of thoracic aortic aneurysm (TAA) is an estimated 5–16 per 100,000; that of abdominal aortic aneurysms (AAA) is 13.5 and that of thoracoabdominal aortic aneurysm (TAAA) is 5.9 (1–6). Aneurysm repair usually involves bridging the aneurysm region with stent grafts in endovascular interventions or open aortic surgery (4, 7–11). However, stent implantation can block orifices of segmental arteries, which results in reduced blood supply to the dorsal parts of the body including the spinal cord. Hence, a dramatic complication of aortic aneurysm treatment is spinal cord ischemia (SCI) (11–15), which in 2.2% leads to paraparesis or paraplegia (16–18).

All blood vessels entering the spinal cord emerge from a single anterior and two, symmetrical posterior spinal arteries (19). These arteries, in turn, originate from the vertebral arteries and descend into the subarachnoid space to the caudal-most segments of the spinal cord (19–21).

Along their way, they are segmentally joined by interspinal arteries, which enter the spinal canal through the intervertebral foramina and accompany the roots of the spinal nerves (22–27). Before entering the subarachnoid space, these arteries also feed a highly sufficient network of anastomoses in the anterior epidural space (28).

The interspinal arteries are branches formed by larger arteries, which segmentally supply blood to the trunk. In the cervical region, they arise from the vertebral artery and in the cranial thorax [thoracic segment (T)1 and T2] from the supreme intercostal artery (22–27). Between T3 and lumbar segment (L)4, they arise from the descending aorta as so-called aortic segmental arteries (ASAs) (19–21, 23, 29). The ASAs of segments T3–T11 are named the posterior intercostal (PIA) arteries; the ASAs of T12 are the subcostal arteries (SCA) and the ASAs in the lumbar region are the lumbar arteries (LA) (29). However, contemporary scientific publications challenge a strict symmetrical origin of 28 ASAs and describe significant variability in numbers, origins, and branching patterns (29–32). However, despite using excellent methodical approaches, these studies do not provide statistically significant information.

Since precise information on ASA topology is key to designing safe surgical approaches, determining the length of stents, and identifying patients with a high risk of suffering SCI during and after aneurysm surgery, we set out to conduct a systematic study of ASA anatomy in a large cohort of human body donors. The aim was to provide detailed information on the topology and variants of the proximal ASA segments and a positional map of their orifices along the aorta.

## 2 Materials and methods

The dorsal intrathoracic and retroperitoneal regions of a total of 200 randomly selected cadavers from body donors were examined. Before death, all body donors had authorized the use of their dead bodies for science and teaching and signed informed consent forms. In addition, the study protocol was approved by the local ethics board (EK Nr: 1748/2021).

All cadavers were perfused via the femoral artery with a solution of 1% formaldehyde/4% carbol for 12–16 h and then immersed in an identical solution for a minimum of 6 months. They had been previously dissected for educational purposes, but care had been taken to spare the retroperitoneal and posterior mediastinal tissues located near the aorta and spine.

Upon gross examination, 47 body donors showed severe aorta pathologies and were excluded. The remaining 153 body donors (73 males, 80 females) with an average age of 81 years (52–99) were included.

The origin and proximal course of the PIA, SCA, and LA were meticulously exposed. The ascending aorta, the aortic arch arteries, the common iliac arteries, and the vessels originating from the descending aorta were then cut and the aorta was extracted. Its anterior wall was removed using the technique described by Shimizu et al. (33).

Digital images of the posterior wall of the opened aortae showing the positions of the ASA orifices were captured with a Canon EOS RP digital camera equipped with an RF 24–105 mm lens (Canon Inc., Tokyo, Japan) and placed on a repro stand (KaiserPRO “RSP,” Kaiser Fototechnik GmbH & Co, Buchen, Germany). An angle ruler (TWL15×30, ATG Kriminaltechnik GmbH, Berlin, Germany) was placed near the specimens. Two Walimex light systems (Niova 150-F Pro, Walser GmbH & Co. KG, Gersthofen, Germany) were employed for bilateral oblique illumination.

**Figure 1:**
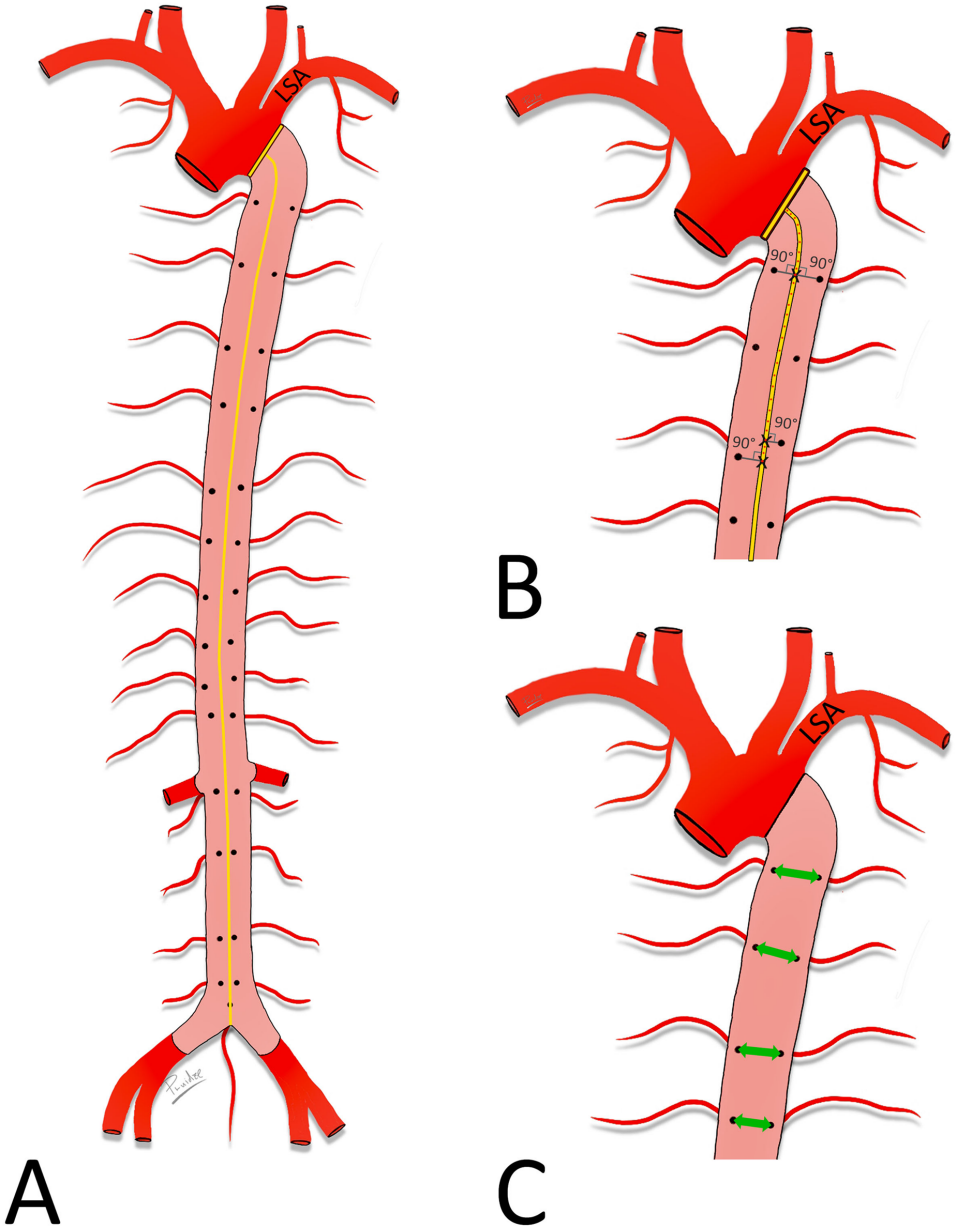
Measured distances. Schematic drawing of the aorta. Posterior wall (pink) of the descending aorta with orifices of the ASA exposed. (**A**) Length of the descending aorta, measured along its centerline (yellow) between the level of the distal edge of the orifice of the LSA and the aorta bifurcation. (**B**) Distances between the distal edge of the orifice of the LSA and the ASA orifices. Note the angle of 90° with the centerline. (**C**) Distances between the right and left ASA orifices (green double-headed arrow).

In the digital images, a centerline was drawn from the level of the distal orifice of the left subclavian artery (LSA) to the aorta bifurcation and its length was measured. The distances between the level of the distal orifice of the left subclavian artery and the level of the origin of each ASA were then measured along the centerline. Finally, the transversal distances between the orifices of symmetrically arising ASAs were measured (Figure 1).

Image J software [National Institutes of Health (NIH), Bethesda, MA, USA], operating on a PC (Microsoft Windows 10Pro, Version 20H2, Washington, DC, USA) was used for the measurements. Microsoft Excel (Microsoft Office LTSC Professional Plus 2021, Version 2108, Microsoft Corporation, USA) and IBM SPSS Statistics Version 29.0 (IBM Corp., Armonk, NY, USA) were used for the descriptive statistics.

## 3 Results

All body donors featured 11 left and right posterior intercostal arteries (PIA 1–11), 1 left and 1 right SCA, and at least 4 proximal left and right segmental lumbar arteries (LA1–4).

### 3.1 Origin and proximal course of the ASAs

**Figure 2:**
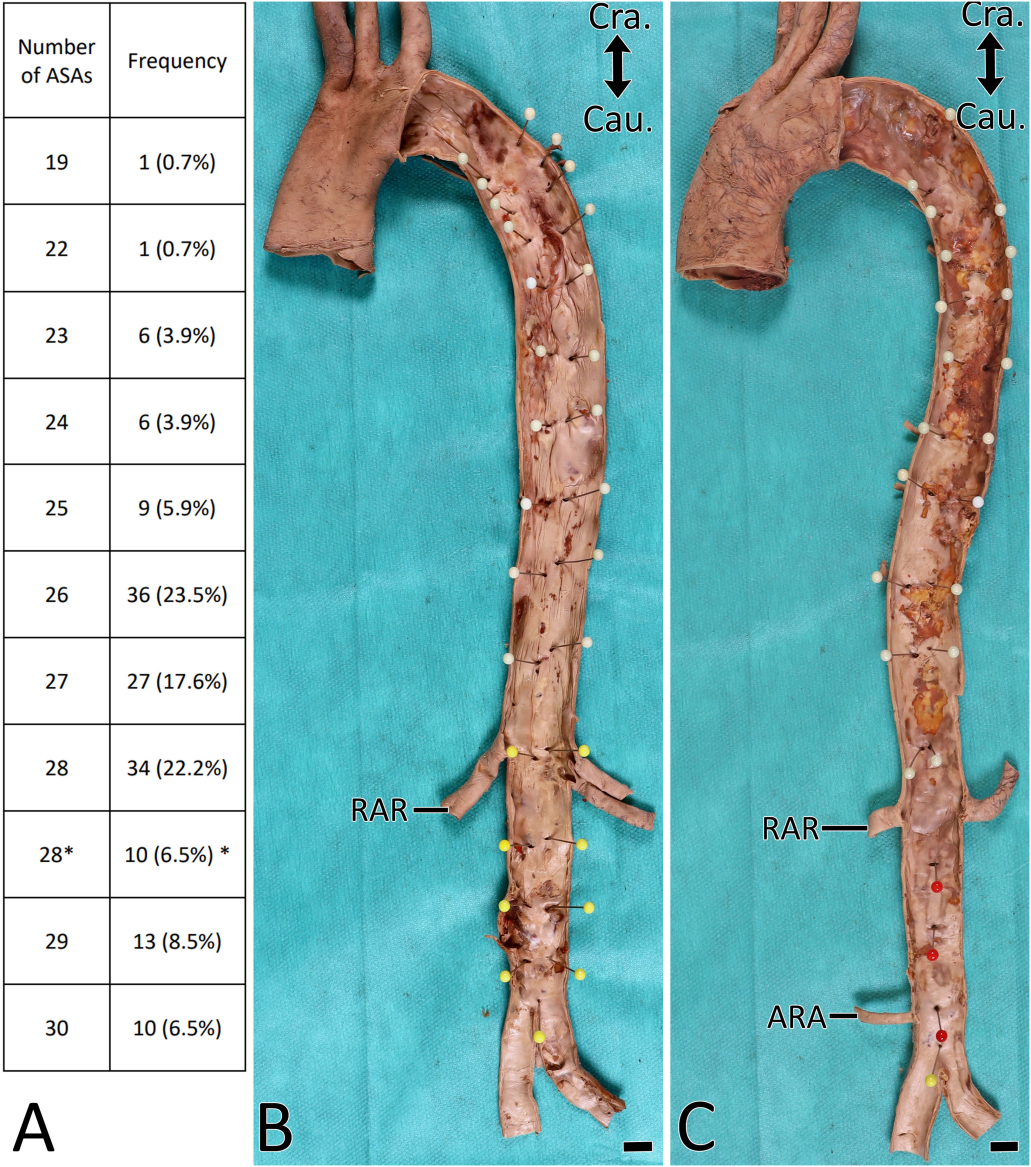
Orifices of the ASA. (**A**) Number of ASA orifices per aorta. Asterisk indicates aortae with strictly segmentally and symmetrically originating T3-L4 ASAs. (**B,C**) Aorta with removed anterior wall. Endoluminal view. ASA orifices labeled with headpins. Note that there are 28 ASA orifices in (**B**) and 23 in (**C**). RAR, right renal artery; ARA, accessory renal artery. Scale bar, 1 cm.

In 152 examined body donors (99.3%) a total of 22–30 ASAs arose from the descending aorta; in one body donor (0.7%) 19 arose and in 107 body donors (69.9%) there were between 26 and 28 (Figure 2).

**Figure 3:**
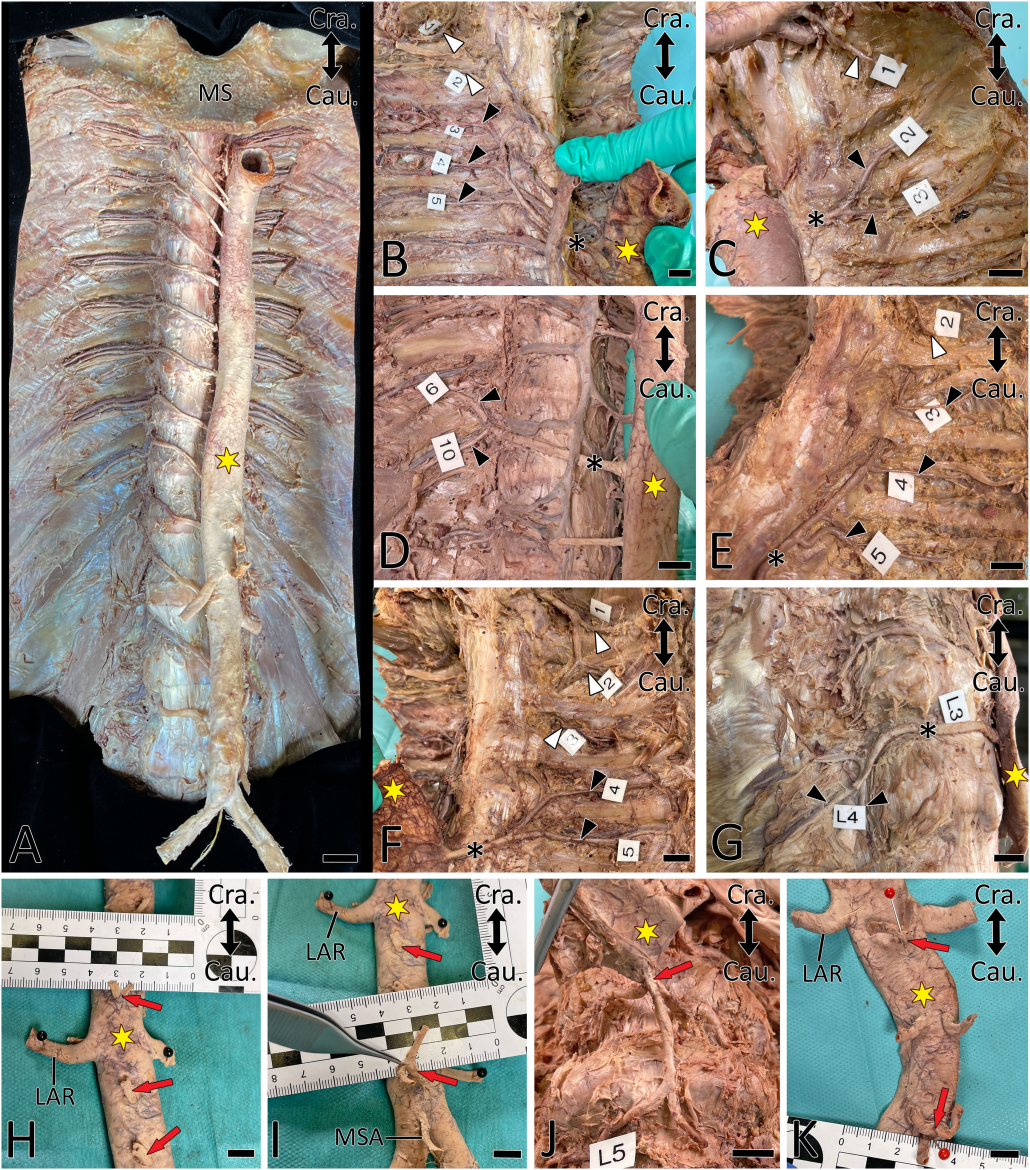
Proximal courses of ASAs. Double-headed arrow indicates cranial (Cra.) and caudal (Cau.). A yellow asterisk indicates the aorta. (**A**) Situs after anatomic exposure of ASAs. (**B–G**) ASA (black asterisk) supplying two (**C,D,F,G**) and three (**B,E**) posterior intercostal arteries (PIA, black arrowhead). White arrowheads indicate PIAs originating from the supreme intercostal artery. Note that PIA3 transits are dorsal to the ribs in (**E**) and (**F**). (**H–K**) Common origin (red arrow) of the right and left segmental arteries. LAR, left renal artery; MSA, median sacral artery; MS, manubrium of sternum. Scale bar, 1 cm.

In 91 body donors (59.5%), the most proximal ASA was PIA3 and in 35 body donors (22.9%) it was PIA2 (Table 1). In an additional 23 (15.0%; 16 unilaterally and 7 bilaterally), PIA2 was a branch arising from PIA3 or PIA4 (Table 2, Figure 3C). In two body donors (1.3%), in whom PIA2 was the first ASA (one on the left and one on the right side), it gave rise to PIA1 (Table 2). In 94 body donors (61.4%), PIA2 arose bilaterally from the supreme intercostal artery. In one body donor (0.7%), the left-sided PIA2 was a direct branch of the vertebral artery.

**Table 1:** Segmental origin patterns of the ASAs in 153 cadaver specimens.

ASA	Bilateral origin of ASA	Unilateral origin of ASA		Common origin of ASA
		Right	Left	
	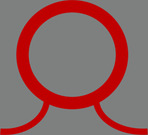	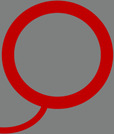	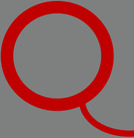	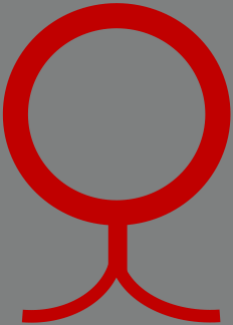
PIA 1	0 (0.0%)	0 (0.0%)	0 (0.0%)	0 (0.0%)
PIA 2	11 (7.2%)	4 (2.6%)	20 (13.1%)	0 (0.0%)
PIA 3	86 (56.2%)	13 (8.5%)	26 (17.0%)	0 (0.0%)
PIA 4	119 (77.8%)	17 (11.1%)	9 (5.9%)	1 (0.7%)
PIA 5	131 (85.6%)	9 (5.9%)	9 (5.9%)	1 (0.7%)
PIA 6	150 (98%)	1 (0.7%)	0 (0.0%)	2 (1.3%)
PIA 7	146 (95.4%)	2 (1.3%)	1 (0.7%)	4 (2.6%)
PIA 8	151 (98.7%)	1 (0.7%)	0 (0.0%)	1 (0.7%)
PIA 9	142 (92.8%)	3 (2.0%)	4 (2.6%)	1 (0.7%)
PIA 10	142 (92.8%)	3 (2.0%)	7 (4.6%)	0 (0.0%)
PIA 11	146 (95.4%)	4 (2.6%)	3 (2.0%)	0 (0.0%)
SCA	152 (99.3%)	1 (0.7%)	0 (0.0%)	0 (0.0%)
LA 1	152 (99.3%)	0 (0.0%)	0 (0.0%)	1 (0.7%)
LA 2	140 (91.5%)	0 (0.0%)	0 (0.0%)	13 (8.5%)
LA 3	139 (90.8%)	0 (0.0%)	0 (0.0%)	14 (9.2%)
LA 4	67 (43.8%)	0 (0.0%)	2 (1.3%)	83 (54.2%)

PIA, posterior intercostal artery; SCA, subcostal artery; LA, lumbar artery.

**Table 2:** ASAs supplying two or more body segments.

Right side										Left side									
PIA 1	1 (0.7)																	1 (0.7)	PIA 1
PIA 2		11 (7.0)	2 (1.3)													4 (3.0)	13 (7.5)		PIA 2
PIA 3				1 (0.7)	40 (26.1)	4 (2.6)	1 (0.7)						6 (3.9)	25 (16.3)	2 (1.3)				PIA 3
PIA 4								10 (6.5)			16 (10.5)	2 (1.3)							PIA 4
PIA 5	10 (6.5)																	9 (5.9)	PIA 5
PIA 6																	1 (0.7)		PIA 6
PIA 7			1 (0.7)													2 (1.3)			PIA 7
PIA 8															1 (0.7)				PIA 8
PIA 9						7 (4.6)								6 (3.9)					PIA 9
PIA 10							8 (5.2)						4 (1.6)						PIA 10
PIA 11								3 (2.0)			4 (1.6)	1 (0.7)							PIA 11
SCA																			SCA
LA 1																			L1
LA 2																			L2
LA 3	3 (2.0)																	1 (0.7)	L3
LA 4																			L4

Blue cells provide the number of cases (percentage in brackets), in which the PIA and SCA originated from the next distal ASA. For example, on the right side PIA3 originates from PIA4 in 26.1%. Red cells provide the number of cases and percentages, in which the segmental arteries originated from the next proximal ASA. Note that in the lumbar region, the LA1–3 always originated segmentally and that three right-sided and one left-sided LA4s were branches of LA3.

In 60 body donors (39.2%; unilaterally in 23.5%), PIA3 branched from a more caudal ASA (Table 2, Figure 3), while in 10 body donors (6.5%), it was a branch of the supreme intercostal artery (Figure 3F). In one body donor (0.7%) the left-sided PIA3 was a branch of the vertebral artery.

In 103 body donors (67.3%), at least one PIA gave rise to an ipsilateral cranial PIA; this was bilateral in 43 body donors (28.1%). In 14 body donors (9.2%), at least 1 PIA gave rise to 2 cranially located PIAs; this was bilateral in 3 body donors (2.0%). In one body donor (0.7%), a right-sided PIA gave rise to three subsequent cranial PIAs. In 97 body donors (63.3%), these variants affected PIA3–PIA6 (153 cases) (Table 2, Figure 3).

In five body donors (3.3%; four left and one right-sided) PIAs branched from more cranially located PIAs (Figure 3G). In one body donor (0.7%) the left SCA arose from PIA11. In another three body donors (2.0%; one bilateral and two right-sided), LA4 arose from LA3 (Table 2, Figure 3G).

In at least one body segment of 86 body donors (56.2%), the left and right-sided ASAs had a common origin (Table 1, Figure 3). One body donor (0.7%) featured this only in the thoracic aorta (Figure 2), 81 (52.9%) only in the abdominal aorta, and four (2.6%) in the thoracic and abdominal aorta. In 15 body donors (9.8%), the ASAs of two or three segments of the abdominal aorta arose in this manner. In 83 body donors (54.2%), the left and right LA4 had a common origin, and in 34 (22.2%) the single vessel, which arose from the aorta, also gave rise to the median sacral artery (MSA). In one case (0.7%), it released both LA4s and both LA5s (Figure 3J).

In three body donors (2.0%) a small local ASA (PIA9, PIA10 on the right, and PIA9 on the left side) branched from the aorta and was joined by a large anastomotic vessel, which arose from a neighboring ASA. In three body donors (2.0%), the right PIA2 was supplied with blood from both the supreme intercostal artery and PIA3.

In 13 body donors (8.5%; 7 unilateral and 3 bilateral), the proximal segments of PIA2 and PIA3 were located dorsally to the ribs (Figures 3E,F).

### 3.2 Distances

**Figure 4:**
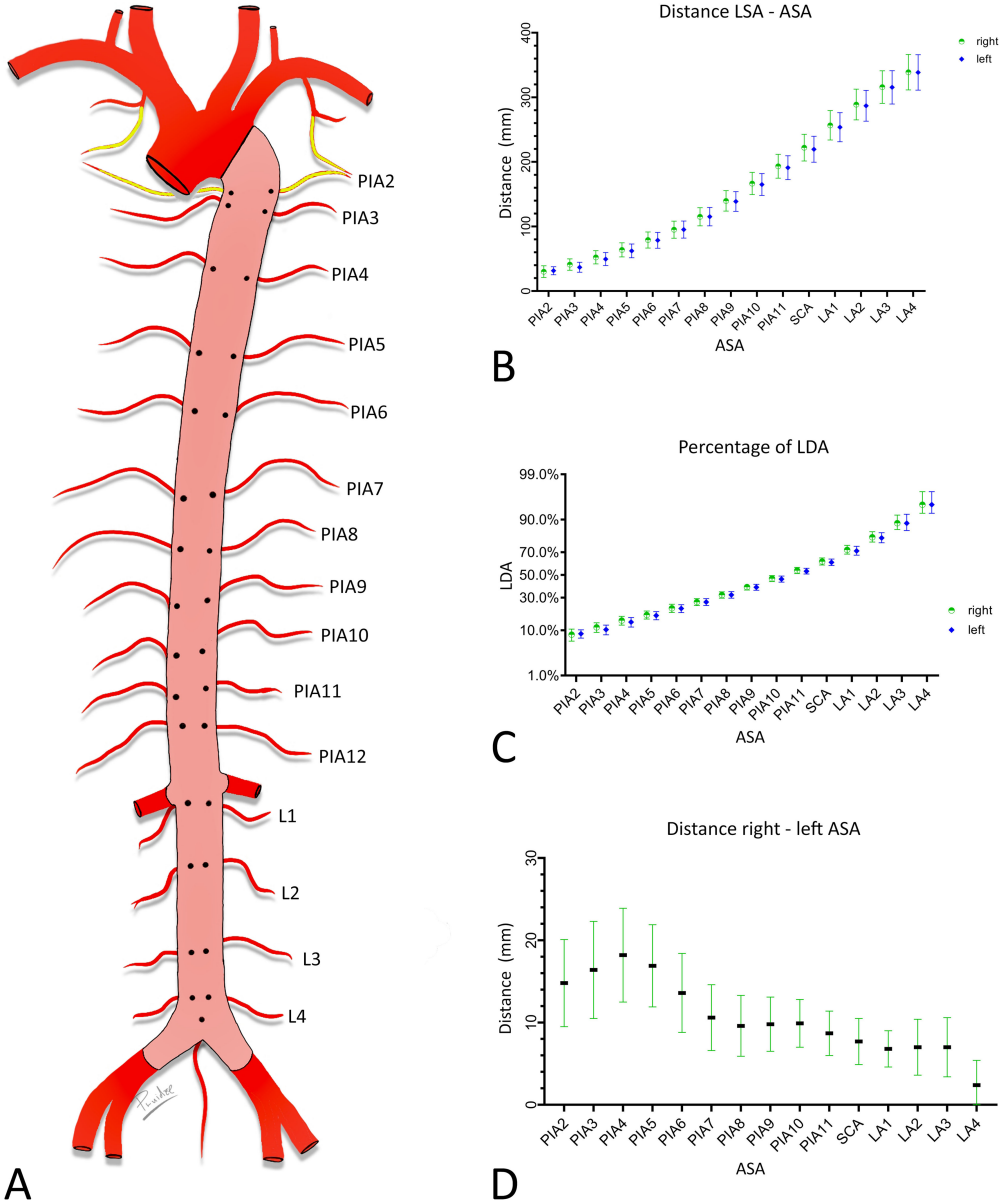
Metric characterization of the positions of the orifices of the ASA. (**A**) Schematic endoluminal view of the descending aorta. (**B,C**) Distance between the level of the origin of the LSA and the level of ASA orifices in mm. (**B**) Ratio of these distances and length of descending aorta in percentages. (**C,D**) Transversal distance between left and right ASAs of the same segment. Graphs provide means and standard deviations. Confidence level = 95%. PIA, posterior intercostal artery; SCA, subcostal artery; LA, lumbar artery.

The length of the descending aorta was 357 mm (±33). The distances between the level of the distal edge of the orifice of the left subclavian artery and the level of the origin of the ASAs, the relationships of these distances with the length of the descending aorta, and the transversal distances between the orifices of the left and right ASAs are presented in Figure 4.

## 4 Discussion

A significant number of studies rely on body donor material to provide information on ASA anatomy (29–32). Since body donor material is precious and its availability is restricted, the studies had to accept relatively small numbers of specimens. This seriously limits the significance of the results and prevents the identification of the full spectrum of variations. Fortunately, we had access to a significant number of randomly selected Caucasian body donors with an average age of 81 years, wherefore we are able to provide statistically valid descriptive and metric data for an elderly Caucasian population.

Most human tissues and organs continuously remodel and with advancing age catabolic processes dominate. Among others, this results in decreased body length and changes in organ topology. In addition, the bodies we used were embalmed using a mixture of formaldehyde and carbol. Such a treatment replaces body liquids and interconnects proteins. This causes soft tissues to shrink up to 20%, particularly if they contain high amounts of water such as loose embryo connective tissue (34–38). The amount of shrinkage of adult blood vessels is much less, but it depends on fixation times, the local environment, and the physical status of the individual. Therefore, no reliable information on the degree of shrinkage of the aorta of elderly body donors can be provided. However, it is certain that the aorta is smaller and shorter than in living patients, while the relationship between the origin of branches remains constant. This has to be taken into consideration when using the provided measurements as a reference in living patients. To relativize the shrinkage effect and to make the measurements provided in this study useful for clinicians, we provide not only the measurements but also relative values—which is, the ratios between the measured distances between ASA orifices and the subclavian artery and the length of the descending aorta.

Our results show that two-thirds of the body donors featured 26–28 ASA orifices and that each body segment between T1 and L4 symmetrically holds segmental arteries. This is in line with descriptions in classical textbooks and earlier studies (19–21, 23, 29). However, in contrast to classical textbooks, we observed symmetrically arising segmental arteries from T3 to L4 in only 6.5%. One specimen even had an as-yet unreported number of a mere 19 ASA orifices. It is evident that patients with such anatomy are likely to suffer a significant reduction in blood supply during interventions. This underscores the relevance of preoperative CT examination of ASA anatomy prior to stent grafting or open aortic surgery. Hence, our data will direct radiologists and interventionists to features worth examining in detail and aid individual decisions on the length of stents, single- or two-step approaches, and intraoperative placement of spinal catheters (11, 39).

Textbooks describe PIA1 and 2 as branches of the supreme intercostal artery, and PIA3 as the most proximal of the descending aorta. Our results show that this arrangement exists in less than one-third of the population. One of the observed variants, PIA2 originating from PIA3, was also recently described by Kocbek and Rakuša (40). PIA2 originating from PIA4, or PIA1 originating from PIA2 are entirely new findings.

Our results demonstrate a high variability in the origin of the ASAs arising between segments T8 and L1. In 17.6%, at least one segmental artery originated from a neighboring ASA. This is of high importance, since the Adamkiewicz artery, which plays a crucial role in spinal cord blood perfusion, emerges from the ASAs of this section of the aorta (41–44). Extensive information on the origin and course of this artery was recently condensed in several studies (41, 43, 44).

As noted previously, the distance between the orifices of symmetrical ASAs decreases from proximal to distal (33). However, our study also revealed the single vessel origin of segmental arteries in the lumbar region and a dramatic increase from the thoraco-lumbar junction toward L4, with the L4 arteries originating in more than 50.0% of the body donors from a single stem. Strikingly, in 22.2%, the median sacral artery also arose from such a common L4 ASA origin. We speculate that this is associated with the processes of aorta remodeling and elongation during late embryogenesis. However, we did not further test this concept, since it is not in the purview of this study.

Our study describes many variants of non-segmental ASA origins that have not yet been described (31, 40, 45, 46). This underscores the necessity of preoperative CT examinations of all thoracic ASAs to avoid reduced blood supply to the spinal cord during the repair of aneurysms in the thoracic aorta (47). Furthermore, it supports the conclusion that careful preoperative imaging for ASA variations and planning a multi-stage stent graft or a physician-modified endograft placement in TAAA reduces the risk of SCI (48). This is demonstrated by various case studies that report preoperatively detected variations that forced the surgeons to change their original treatment strategy (49–53).

In one body donor, PIA2 and PIA3 arose directly from the vertebral artery. Such a situation has been previously described (31, 54, 55). Other previously described rare variations are an anastomosis between the supreme intercostal artery and one or two distally located ASAs (31) and PIAs, which cross the ribs dorsally (29, 40). Here, our data are the first that show that these variations are restricted to the cranial thorax.

The ASA variations we identified in a Central European population align with other ASA variations described in other populations, such as Asians and Americans (31, 33). However, in a Chinese population, a variation was reported by Jie et al. (56) that was not detected in our study. This variation was all the left and right 4th to 11th PIAs originating from an arterial trunk, arising at the level T12/L1 from the aorta. However, this variation was also detected in a Central European population (57). This hints that there could be no differences between ethnic groups.

In summary, the results of our comprehensive and systematic large-scale study provide new anatomic maps, metric data, and statistics on the large spectrum of variants of the origin and proximal course of the ASAs. The results assist in the interpretation of preoperative CT assessment for avoiding spinal cord ischemia during the repair of pathologies of the descending aorta and can serve as a sound fundament for deciding on single- or two-step interventions to repair aorta aneurysms, selecting correct stent lengths during endovascular aortic interventions, and managing endovascular and open aorta surgery.

## Data Availability

The original contributions presented in the study are included in the article/Supplementary Material, further inquiries can be directed to the corresponding author.
