# Supplementary material for: Anatomy of the aortic segmental arteries—the fundamentals of preventing spinal cord ischemia in aortic aneurysm repair
Source: Front Cardiovasc Med. 2024 Dec 3;11:1475084. doi: 10.3389/fcvm.2024.1475084 (PMC11649645; doi:10.3389/fcvm.2024.1475084)
Supplement: Supplementary file 1 [file Table1.docx]

**Supplementary Table 1:** Distances between lower edge of origin of left subclavian artery and orifices of segmental arteries. PIA, posterior intercostal artery; SC, subcostal artery; LA, lumbar artery; L, left; R, right, n, number of specimens having the right and left respective orifice.

| **ASA** | **side** | **n** | **mean**  **(mm)** | **median**  **(mm)** | **standard deviation** | **minimum**  **(mm)** | **maximum**  **(mm)** |
| --- | --- | --- | --- | --- | --- | --- | --- |
| **PIA2** | R | 15 | 30 | 30 | 9 | 16 | 50 |
|  | L | 31 | 31 | 32 | 6 | 20 | 41 |
| **PIA3** | R | 99 | 41 | 40 | 9 | 17 | 66 |
|  | L | 112 | 37 | 36 | 8 | 20 | 58 |
| **PIA4** | R | 137 | 52 | 52 | 10 | 29 | 83 |
|  | L | 129 | 50 | 49 | 10 | 29 | 83 |
| **PIA5** | R | 141 | 64 | 63 | 11 | 41 | 98 |
|  | L | 141 | 62 | 61 | 11 | 38 | 96 |
| **PIA6** | R | 153 | 79 | 78 | 12 | 46 | 112 |
|  | L | 152 | 79 | 78 | 12 | 44 | 114 |
| **PIA7** | R | 152 | 95 | 94 | 13 | 61 | 131 |
|  | L | 151 | 95 | 94 | 13 | 61 | 131 |
| **PIA8** | R | 153 | 115 | 115 | 14 | 79 | 159 |
|  | L | 152 | 115 | 115 | 14 | 81 | 159 |
| **PIA9** | R | 146 | 140 | 141 | 16 | 102 | 197 |
|  | L | 147 | 139 | 140 | 15 | 102 | 197 |
| **PIA10** | R | 145 | 167 | 167 | 17 | 128 | 231 |
|  | L | 149 | 165 | 163 | 17 | 125 | 231 |
| **PIA11** | R | 150 | 193 | 192 | 18 | 155 | 261 |
|  | L | 149 | 191 | 190 | 18 | 155 | 258 |
| **SCA** | R | 153 | 222 | 220 | 20 | 182 | 297 |
|  | L | 152 | 219 | 218 | 20 | 178 | 290 |
| **LA1** | R | 153 | 257 | 256 | 23 | 209 | 332 |
|  | L | 153 | 254 | 253 | 23 | 191 | 327 |
| **LA2** | R | 153 | 289 | 290 | 24 | 237 | 369 |
|  | L | 153 | 287 | 287 | 24 | 236 | 366 |
| **LA3** | R | 153 | 316 | 317 | 25 | 257 | 400 |
|  | L | 153 | 316 | 315 | 26 | 257 | 398 |
| **LA4** | R | 150 | 339 | 339 | 27 | 276 | 424 |
|  | L | 152 | 339 | 339 | 28 | 276 | 426 |

**Supplementary Table 2:** Distance between level of ASA in relation to length of descending aorta. PIA, posterior intercostal artery; SCA, subcostal artery; LA, lumbar artery; n, number of specimens having the respective orifice.

| **ASA** | **side** | **n** | **mean**  **(%)** | **median**  **(%)** | **standard deviation** | **minimum**  **(%)** | **maximum**  **(%)** |
| --- | --- | --- | --- | --- | --- | --- | --- |
| **PIA2** | R | 15 | 8.4 | 8.8 | 2.1 | 4 | 13 |
|  | L | 31 | 8.8 | 8.7 | 1.5 | 6 | 12 |
| **PIA3** | R | 99 | 11.3 | 11.3 | 2.1 | 6 | 18 |
|  | L | 112 | 10.3 | 10.1 | 1.9 | 6 | 16 |
| **PIA4** | R | 137 | 14.6 | 14.6 | 2.3 | 8 | 22 |
|  | L | 129 | 13.8 | 13.9 | 2.4 | 8 | 20 |
| **PIA5** | R | 141 | 17.8 | 17.8 | 2.4 | 12 | 27 |
|  | L | 141 | 17.4 | 17.2 | 2.3 | 12 | 26 |
| **PIA6** | R | 153 | 22.1 | 22.2 | 2.7 | 15 | 30 |
|  | L | 152 | 22.0 | 21.9 | 2.7 | 14 | 30 |
| **PIA7** | R | 152 | 26.6 | 26.5 | 2.6 | 20 | 35 |
|  | L | 151 | 26.6 | 26.5 | 2.6 | 20 | 35 |
| **PIA8** | R | 153 | 32.2 | 32.2 | 2.6 | 25 | 43 |
|  | L | 152 | 32.3 | 32.2 | 2.7 | 25 | 43 |
| **PIA9** | R | 146 | 39.0 | 39.2 | 2.6 | 31 | 45 |
|  | L | 147 | 38.8 | 38.8 | 2.6 | 31 | 45 |
| **PIA10** | R | 145 | 46.7 | 46.8 | 2.7 | 39 | 53 |
|  | L | 149 | 46.2 | 46.4 | 2.8 | 39 | 53 |
| **PIA11** | R | 150 | 54.0 | 54.2 | 2.7 | 46 | 60 |
|  | L | 149 | 53.5 | 53.6 | 2.6 | 46 | 59 |
| **SCA** | R | 153 | 62.2 | 62.3 | 3.1 | 54 | 71 |
|  | L | 152 | 61.5 | 61.5 | 2.8 | 53 | 68 |
| **LA1** | R | 153 | 71.9 | 72.3 | 3.4 | 61 | 79 |
|  | L | 153 | 71.1 | 71.6 | 3.5 | 61 | 79 |
| **LA2** | R | 153 | 80.9 | 81.1 | 3.2 | 69 | 88 |
|  | L | 153 | 80.4 | 80.7 | 3.2 | 68 | 87 |
| **LA3** | R | 153 | 88.5 | 88.5 | 3.6 | 77 | 94 |
|  | L | 153 | 88.4 | 88.5 | 3.6 | 77 | 94 |
| **LA4** | R | 150 | 94.8 | 94.7 | 2.5 | 84 | 99 |
|  | L | 152 | 94.8 | 94.7 | 2.5 | 84 | 99 |

**Supplementary Table 3:** Distances between right and left orifices of segmental arteries. PIA, posterior intercostal artery; SC, subcostal artery; LA, lumbar artery; n, number of specimens having the respective orifice.

| **ASA** | **n** | **mean**  **(mm)** | **median**  **(mm)** | **standard deviation** | **minimum**  **(mm)** | **maximum**  **(mm)** |
| --- | --- | --- | --- | --- | --- | --- |
| **PIA2** | 10 | 15 | 17 | 5 | 8 | 24 |
| **PIA3** | 86 | 16 | 16 | 6 | 3 | 31 |
| **PIA4** | 124 | 18 | 18 | 6 | 0 | 34 |
| **PIA5** | 134 | 17 | 17 | 5 | 0 | 31 |
| **PIA6** | 152 | 14 | 14 | 5 | 0 | 30 |
| **PIA7** | 150 | 11 | 10 | 4 | 0 | 22 |
| **PIA8** | 152 | 10 | 9 | 4 | 0 | 34 |
| **PIA9** | 144 | 10 | 9 | 3 | 0 | 28 |
| **PIA10** | 143 | 10 | 9 | 3 | 6 | 23 |
| **PIA11** | 146 | 9 | 8 | 3 | 4 | 18 |
| **SCA** | 152 | 8 | 7 | 3 | 0 | 23 |
| **LA1** | 153 | 7 | 7 | 2 | 0 | 13 |
| **LA2** | 153 | 7 | 7 | 3 | 0 | 18 |
| **LA3** | 153 | 7 | 7 | 4 | 0 | 17 |
| **LA4** | 150 | 2 | 0 | 3 | 0 | 11 |
